# Developmental Programmed Cell Death Involved in Ontogenesis of *Dictamnus dasycarpus* Capitate Glandular Hairs

**DOI:** 10.3390/plants12020395

**Published:** 2023-01-14

**Authors:** Yafu Zhou, Gen Li, Guijun Han, Lulu Xun, Shaoli Mao, Luyao Yang, Yanwen Wang

**Affiliations:** 1 Xi’an Botanical Garden of Shaanxi Province, Institute of Botany of Shaanxi Province, 17 Cui Hua Nan Road, Xi’an 710061, China; 2 Shaanxi Engineering Research Centre for Conservation and Utilization of Botanical Resources, 17 Cui Hua Nan Road, Xi’an 710061, China

**Keywords:** programmed cell death (PCD), capitate glandular hairs, formation, essential oils, *Dictamnus dasycarpus*

## Abstract

Plant glandular trichomes have received much attention due to their commercial and biological value. Recent studies have focused on the development of various glands in plants, suggesting that programmed cell death (PCD) may play an important role during the development of plant secretory structures. However, the development processes and cytological characteristics in different types of plant secretory structures differed significantly. This study aims to provide new data on the developmental PCD of the capitate glandular hairs in *Dictamnus dasycarpus*. Light, scanning, immunofluorescence labeling, and transmission electron microscopy were used to determine the different developmental processes of the capitate glandular hairs from a cytological perspective. Morphologically, the capitate glandular hair originates from one initial epidermal cell and differentiates into a multicellular trichome characterized by two basal cells, two lines of stalk cells, and a multicellular head. It is also histochemically detected by essential oils. TUNEL-positive reactions identified nuclei with diffused fluorescence or an irregular figure by DAPI, and Evans blue staining showed that the head and stalk cells lost their viability. Ultrastructural evidence revealed the developmental process by two possible modes of PCD. Non-autolytic PCD was characterized by buckling cell walls and degenerated nuclei, mitochondria, plastids, multivesicular body (MVB), and end-expanded endoplasmic reticulum in the condensed cytoplasm, which were mainly observed in the head cells. The MVB was detected in the degraded vacuole, a degraded nucleus with condensed chromatin and diffused membrane, and eventual loss of the vacuole membrane integrity exhibited typical evidence of vacuole-mediated autolytic PCD in the stalk cells. Furthermore, protoplasm degeneration coupled with dark oil droplets and numerous micro-dark osmiophilic substances was observed during late stages. The secretion mode of essential oils is also described in this paper.

## 1. Introduction

The glandular trichome is a kind of secretory structure on the aerial surfaces of plants that can secrete diverse secondary products as well as other lipophilic and non-lipophilic compounds [1]. As a result, the plants’ secretions have received great interest due to their commercial and biological values.

Early studies indicated that the synthesis and accumulation of secretions are followed by degeneration and autolysis in the secretory cells, including the autolysis of glandular cells, nuclear disorganization, cytoplasmic condensation, and the disruption of endoplasmic reticulum (ER) cisternae and multivesicular bodies [2,3,4,5,6,7]. Amrehn and Spring [8] reported that the deposition of fluorescent and brownish metabolites in the mature secretory cells of sunflower linear glandular trichomes is associated with the disintegration of their subcellular compartments. Recently, a comparative proteomic analysis of latex from *Euphorbia kansui* laticifers at different developmental stages was studied by Zhao et al. [9], and the result showed that partial cytoplasmic degradation is positively correlated with secondary metabolite synthesis. Dangl et al. [10] believed that glandular cell autolysis with the release of secretory products might be a PCD phenomenon. However, few studies at the time directly investigated the programmed cell death (PCD) of plant glands. 

A study by Gaffal et al. [11] confirmed that PCD occurred in the floral nectary of *Digitalis purpurea,* and the authors provided ultrastructural evidence. Recent studies have focused on the development of the cotton pigment gland [12], secretory cavities in the fruits of *Citrus sinensis* [13,14] and *Dictamnus dasycarpus* leaves [15], floral nectary senescence in *Ipomoea purpurea* [16], *Decaisnea fargesii* laticiferous canal [17], *Araucaria angustifolia* mucilage cells [18], *Tillandsia* trichomes [19], and the trichome-like cavities of *D. dasycarpus* [20], suggesting that PCD may play important roles in the development of plant secretory structures. However, the development process and cytological characteristics in different types of plant secretory structures differed significantly.

Our previous studies indicated that the synthesis and accumulation of essential oils in the *D. dasycarpus* secretory cavities showed a connection with secretory cell autolysis, which was proven to be a PCD process [15]. Although molecular methods have been widely used to investigate PCD in plants, studies on the PCD process of plant glandular hairs are inadequately addressed due to their small size and complex chemical composition. It is necessary to reveal the development process of plant secretory structures from a cytological point of view, to identify different types of cell death, and to provide essential information for cell death research. The present study aims to provide new data on the developmental PCD by subjecting the *D. dasycarpus* glandular hairs to TUNEL and DAPI assay, as well as viability and cellular ultrastructure using light, scanning, immunofluorescence labeling, and transmission electron microscopy.

## 2. Results

### 2.1. Morphology and Distribution

The *D. dasycarpus* capitate glandular hairs are located on the vegetative and reproductive organs, and are interlaced with non-glandular hairs and trichome-like cavities on the young stems, floral axes, sepals, filaments, and ovaries of the plant. The capitate glandular hairs only occur on the leaf axial surfaces above the veins.

**Figure 1:**
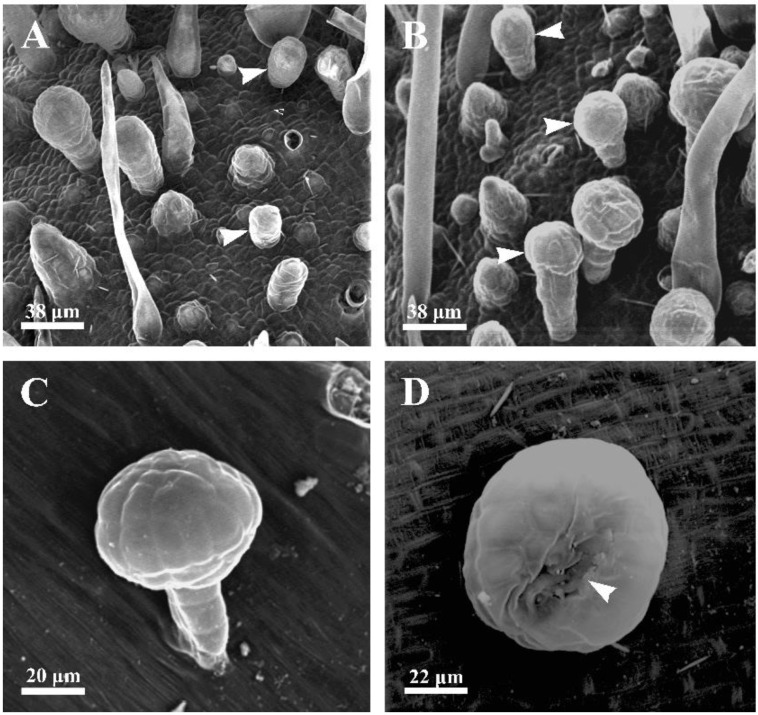
Scanning electron micrographs (SEM) of the capitate glandular hairs located on the inflorescence. (**A**) Late stage 1, showing the cylindrical shape of capitate glandular hairs (arrowheads). (**B**) Early stage 2, showing capitate glandular hairs with multicellular head (arrowheads). (**C**) Late stage 3 or stage 4, showing glandular hairs with a two-lined stalk and multicellular head. (**D**) Stage 4, showing the degraded area (arrowhead) occurring in the head of the capitate glandular hair, through which the secretion could be released during the late stages.

**Figure 2:**
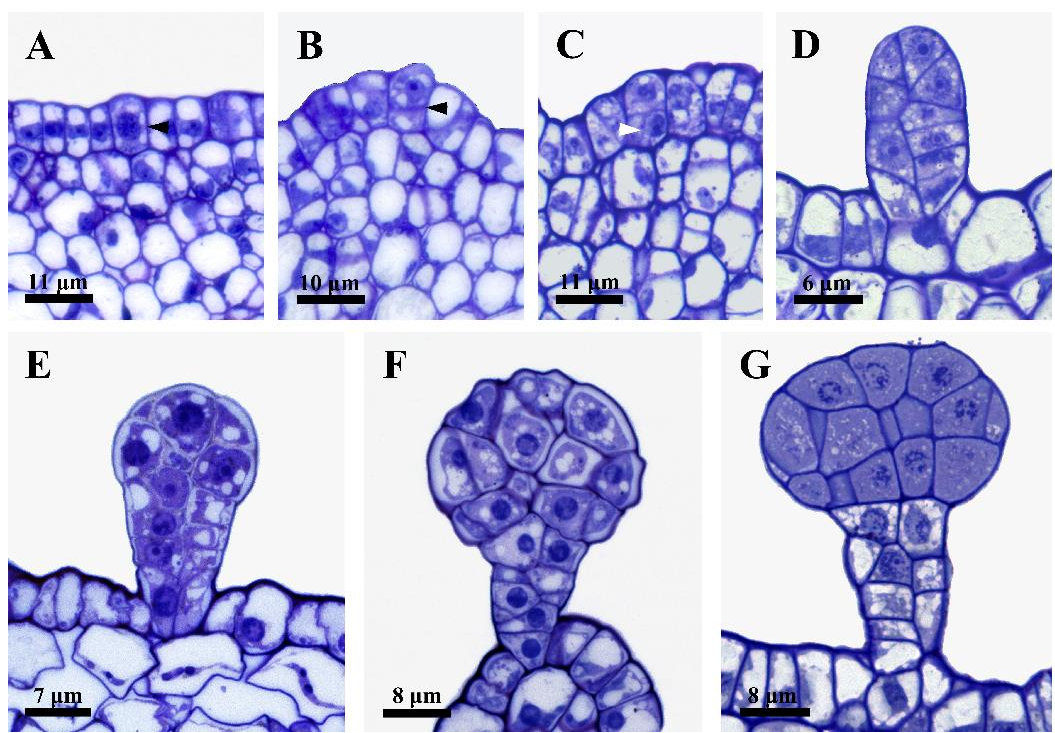
Light microscopic observations of the capitate glandular hairs. (**A**–**D**) Stage 1. (**A**) The initial cell with dense cytoplasm and large nuclei. (**B**) Two sister cells from one initial cell on the epidermis (arrowhead). (**C**) Anticlinal division of the lower cell (arrowhead). (**D**) A cylindrical shape with several cells formed. (**E**,**F**) Stage 2. (**E**) A multicellular head was formed. (**F**) Head and stalk of the capitate glandular hair. (**G**) Stage 3: condensed cytoplasm occurred in the multicellular head.

The early developmental stage of the capitate glandular hairs in *D. dasycarpus* is characterized by a cylindrical shape (Figure 1A). The fully developed capitate glandular hairs consist of two basal cells, two lines of stalk cells, and a large multicellular head with cutinized lateral walls (Figure 1B,C and Figure 2F–G). Scanning electron microscopy observation revealed a depression area in the gland’s upper-center head at the late degeneration stage (Figure 1D).

### 2.2. Development of Capitate Glandular Hairs

**Figure 3:**
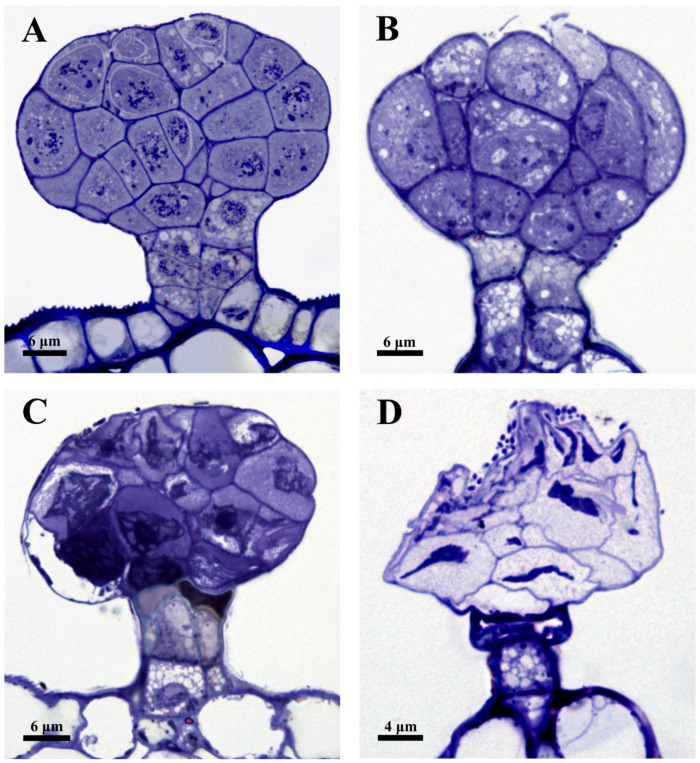
Light microscopic observations of the capitate glandular hairs. (**A**) Late stage 3, condensed cytoplasm occurred in the multicellular head and stalk cells with disorganized cytoplasms and degraded nuclei. (**B**–**D**) Stage 4. (**B**) Early stage 4, showing degraded cytoplasms and cuticles on the top of the head cells, condensed cytoplasms of the lower head cells in capitate glandular hairs, and much more cutinization of the stalk cells full of brown osmiophilic substances. (**C**) Late stage 4, disorganized membrane systems and dark or brown osmiophilic substances in the head and stalk cells, as well as disruption of the head cells and cell wall thickening in the stalk cells. (**D**) Late stage 4, showing empty head cells with little residue and degenerated cell walls, and cell wall thickening in the stalk cells.

To evaluate the ontogenesis of the glandular hairs, four different stages were subdivided based on the microscopic observations: stage 1, initial stage—the glandular hair from one initial epidermal cell differentiated into a multicellular cylindrical shape (Figure 2A–D); stage 2, fully developed stage—an evident head formed on the glandular hair (Figure 2E,F); stage 3, early degeneration stage—the head cells of the glandular hair showed condensed cytoplasms (Figure 2G and Figure 3A); stage 4, late degeneration stage—the protoplast, cell walls, and cuticle were obviously degenerated (Figure 3B–D).

#### 2.2.1. Stage 1: Initial Stage

The glandular hair originates from the epidermal cell (Figure 2A), which divides into two sister cells by periclinal division (Figure 2B): the lower cell divides into two bottom cells by anticlinal division (Figure 2C), and the upper cell eventually differentiates into a multicellular cylindrical head and stalk. The secretory cells were characterized by dense cytoplasms and large nuclei, as well as small vacuoles, during this stage (Figure 2A–D).

#### 2.2.2. Stage 2: Fully Developed Stage

With differentiation, the capitate glandular hair was distinguished by a multicellular head and stalk formed by several divisions of the upper cell; concurrently, the volume of the head increased and the small vacuoles of the head cells fused to form larger vacuoles. However, no evident vacuolization of the cytoplasm was observed in the head cells, which were characterized by large nuclei and dense cytoplasms (Figure 2E). Finally, during late stage 2, the number of head cells reached the maximum, and the anticlinal walls of the stalk and the head cells of the gland were cutinized (Figure 2F).

#### 2.2.3. Stage 3: Early Degeneration Stage

The glandular hair head cells were characterized by remarkably condensed cytoplasms and degenerated nuclei. The contrast between the cytoplasms and the nuclei was low and difficult to distinguish, and some of the nuclei almost disappeared (Figure 2G), while the protoplasts of the stalk cells showed misshapen nuclei and significant vacuolization (Figure 2G). Late in this stage, both head and stalk cells were detected by observing the condensed cytoplasms and diffused nuclei with condensed chromatin (Figure 3A).

#### 2.2.4. Stage 4: Late Degeneration Stage

Notably, remarkable autolysis of the degenerated cytoplasms and cuticles on the top head cells was firstly observed, leaving the degraded cells with numerous irregular flocculent structures (Figure 3B), and the tightly arranged head cells from the early stage loosened (Figure 3B). As this process continued, the protoplasts of the secretory cells gradually degraded; the walls of the head cells degenerated to a thinner shape, but showed significant thickening in the stalk cells (Figure 3C,D); and a brown or darkly stained substance with a disorganized membrane system could be observed in the head and stalk cells (Figure 3C), leaving a small amount of residual substances in the deformed cells with thinner and degraded walls in the head cells (Figure 3D).

### 2.3. TUNEL and DAPI Assay

**Figure 4:**
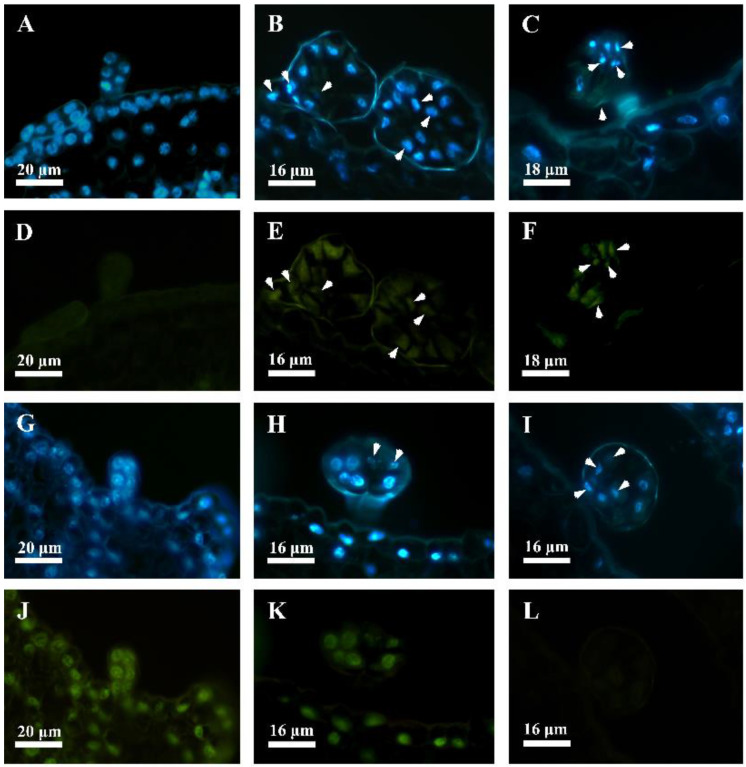
Nuclear DNA fragmentation of the capitate glandular hairs of *D. dasycarpus* in the developmental process and control. (**A**) DAPI-positive nuclei of the glandular hair at stage 1. (**B**) Diffused fluorescence or misshapen nuclei of the glandular hair at stage 3 (arrowheads). (**C**) Condensed and moon-shaped nuclei at early stage 4 (arrowheads). (**D**) TUNEL-negative nuclei at stage 1. (**E**) TUNEL-positive reaction of the capitate glandular hair (arrowheads) with no labeling on the other tissue at stage 3. (**F**) Condensed nuclei with TUNEL-positive reaction (arrowheads) at early stage 4. (**G**) DAPI-stained glandular hair in control of stage 1. (**H**) DAPI-stained glandular hair in control of early stage 3, showing diffused fluorescence or misshapen nuclei (arrowheads). (**I**) DAPI-stained glandular hair in control of late stage 3, showing more diffused fluorescence or misshapen nuclei in most of secretory cells of the glandular hair (arrowheads). (**J**,**K**) TUNEL-positive controls at stage 1 and early stage 3, showing the TUNEL-positive reactions of the capitate glandular hair and the other tissue. (**L**) TUNEL-negative control at late stage 3, showing the TUNEL-negative reactions of the capitate glandular hair and the other tissue.

A further analysis of nuclear DNA degradation and nuclear changes during the ontogenesis of the capitate glandular hair was conducted using a TUNEL assay and DAPI counterstaining (Figure 4). In the earliest stage, the large nuclei of the capitate glandular hair were detected by a DAPI-positive (Figure 4A) and TUNEL-negative reaction (Figure 4D). At stage 3, some of the nuclei detected by diffused fluorescence, or those with misshapen shapes according to DAPI staining (Figure 4B), were TUNEL-positive (Figure 4E), while other parenchyma cells were TUNEL-negative (Figure 4E). At early stage 4, most of the nuclei completely degraded, and the remaining cells with DAPI-labeled nuclei were characterized by an irregular figure (Figure 4C) detected by a TUNEL-positive reaction with slight fluorescence (Figure 4F). TUNEL and DAPI-positive controls were conducted; the results showed that DAPI-positive cells at stage 1 (Figure 4G) and early stage 3 (Figure 4H) were detected by TUNEL-positive reactions exclusively in the capitate glandular hair and other tissues (Figure 4J,K). The negative control showed that some of the nuclei detected by diffused fluorescence by DAPI staining (Figure 4I) were detected by a TUNEL-negative reaction in the capitate glandular hair at late stage 3 (Figure 4L).

### 2.4. Viability Staining—Evans Blue

**Figure 5:**
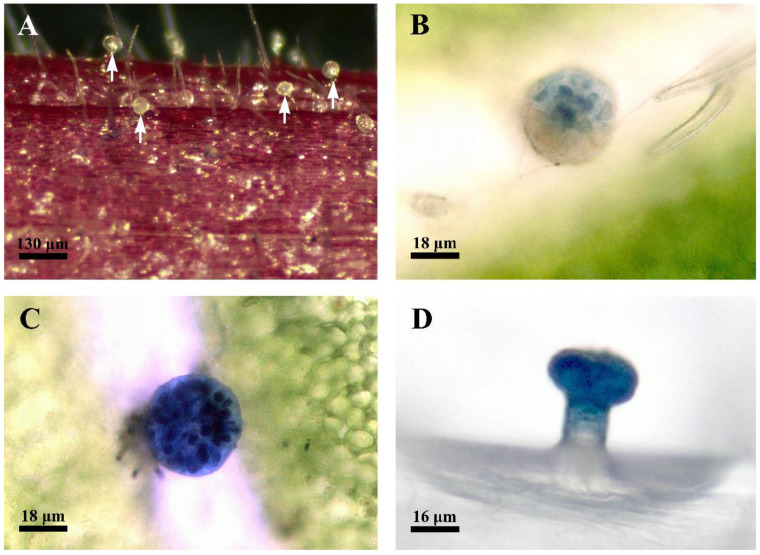
Viability staining with Evans blue of the glandular hair. (**A**) Early stage, negative reaction of the glandular hair with Evans blue (arrowheads) on the floral axis. (**B**) Late stage 3 or early stage 4, part of the secretory cells of the glandular hair stained positive with Evans blue on the leaf. (**C**) Late stage 4, positive reaction of the head of the glandular hair with Evans blue on the leaf. (**D**) Late stage 4, positive reactions of the glandular hair head and stem, as well as negative reaction of the filament, with Evans blue.

The plant protoplast always showed a loss of membrane integrity and, consequently, viability during the PCD process. To further elucidate the viability of the secretory cells, we used Evans blue staining to identify the developing glandular hair of *D. dasycarpus*. The results indicated that the glandular hair, at the early stage, showed a negative reaction with Evans blue stains (Figure 5A). During late stage 3 or early stage 4, parts of the secretory cells of the glandular hair were stained positive with Evans blue (Figure 5B), and all of the head and stalk cells of the gland almost showed Evans blue positive reactions at late stage 4 (Figure 5C,D). Meanwhile, the leaf epidermal cells (Figure 5B,C) and the filament (Figure 5D) were negative with Evans blue stains.

### 2.5. Ultrastructural Changes in the Capitate Glandular Hairs

In order to characterize the ultrastructural changes consistent with the plant PCD and secretion mechanism, the ultrastructural changes in the different developmental stages of the capitate glandular hair in *D. dasycarpus* were detected by transmission electron microscopy.

#### 2.5.1. Stage 1: Origin Stage

**Figure 6:**
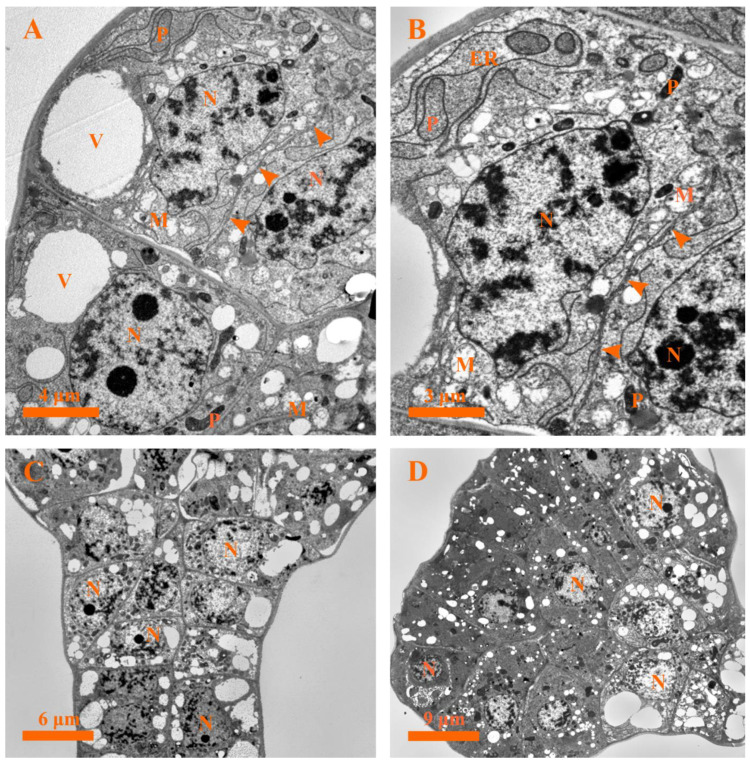
Ultrastructure of the capitate glandular hair in *D. dasycarpus.* (**A**) Stage 1, characteristics of the secretory cells included large nuclei, dense cytoplasms, abundant plastids and mitochondria, evident endoplasmic reticula, and division of the secretory cells (arrowheads). (**B**) Stage 1, details of mitochondria, plastids, endoplasmic reticula, large nuclei with evident chromatin, and division of the secretory cells (arrowheads). (**C**) Stage 2, bigger vacuoles and small ones fused with each other in the stalk cells. (**D**) Stage 3, condensed cytoplasms in the head cells of the capitate glandular hair. ER, endoplasmic reticulum; M, mitochondria; N, nucleus; P, plastid; V, vacuole.

During stage 1, the secretory cells showed characteristics of meristematic cells that possessed chromatin, large and rounded nuclei with distinct nucleoli, dense cytoplasms, abundant plastids and mitochondria, endoplasmic reticula, small vacuoles, and insignificant amounts of osmiophilic material. Division of the head cell was also observed (Figure 6A,B).

#### 2.5.2. Stage 2: Fully Developed Stage

At stage 2, a head formed, and the amount of secretory cells in the capitate glandular hair reached the maximum. No evident vacuolization was observed in the head cells during the entire developmental process (Figure 6C). In the stalk cells, the small vacuoles tended to fuse to each other, increasing their dimensions in this stage. However, the volumes of the nuclei remained large, and some of the nucleoli could also be observed in the secretory cells (Figure 6C).

#### 2.5.3. Stage 3: Early Degeneration Stage

**Figure 7:**
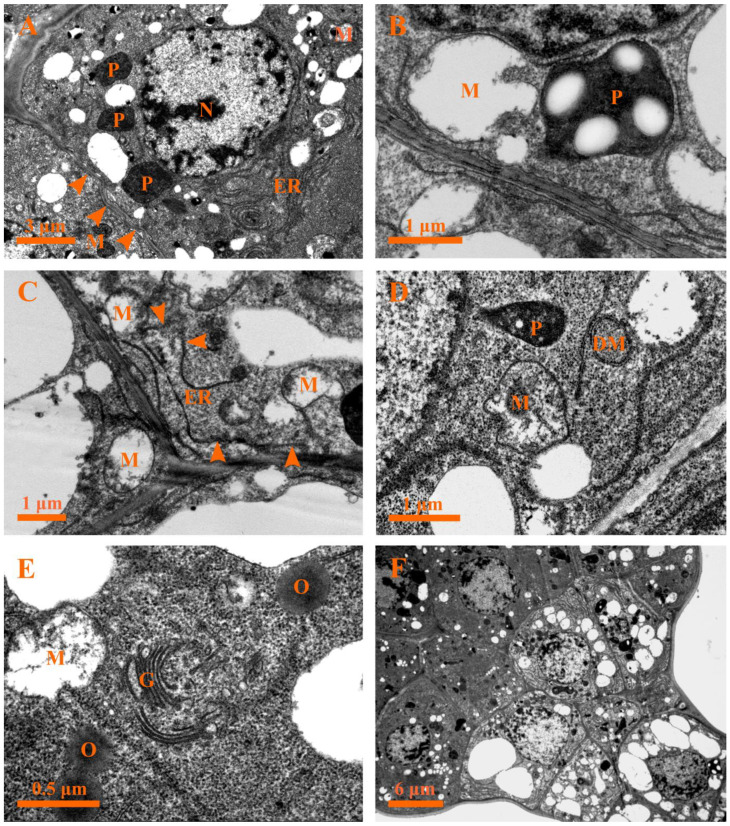
Ultrastructure of the capitate glandular hair in *D. dasycarpus*. (**A**) Stage 3, head cells with condensed plastids, degraded mitochondria, buckling of cell walls with wavy shape (arrowheads), and deformed nuclei with condensed chromatin close to the nuclear membrane. (**B**) Stage 3, detailed deformed mitochondria with degraded membranes, plastids with condensed matrices, and abundant endoplasmic reticula near the plasma membrane. (**C**) Stage 3, degraded endoplasmic reticulum with a diffused end (arrowheads). (**D**) Stage 3, misshapen mitochondria, condensed plastids, and double membrane-bounded structures. (**E**) Stage 3, vesicles from Golgi bodies, degrade mitochondria, and oil droplets. (**F**) Stage 3, condensed cytoplasms of the head cells and vacuolization of the stalk cells. DM, double membrane-bounded structure; ER, endoplasmic reticulum; G, Golgi body; M, mitochondria; N, nucleus; O, oil droplet; P, plastid.

Early in this stage, drastic changes occurred in the secretory cells: for example, the head cells were characterized by condensed cytoplasm (Figure 6D), buckling of the cell walls along with the formation of a wavy shape was observed, and a deformed nucleus with condensed chromatin close to the nuclear membrane could be seen (Figure 7A). Furthermore, degraded plastids with condensed matrices, deformed mitochondria with degraded membranes, endoplasmic reticula with the diffused ends near the plasma membranes, and double membrane-bounded structures were observed (Figure 7B–D). In addition, the Golgi bodies secreted several vesicles which tended to rupture in the cytoplasm (Figure 7E). Moreover, the stalk cells were identified by large vacuoles and evident nuclei with condensed chromatin (Figure 7F).

**Figure 8:**
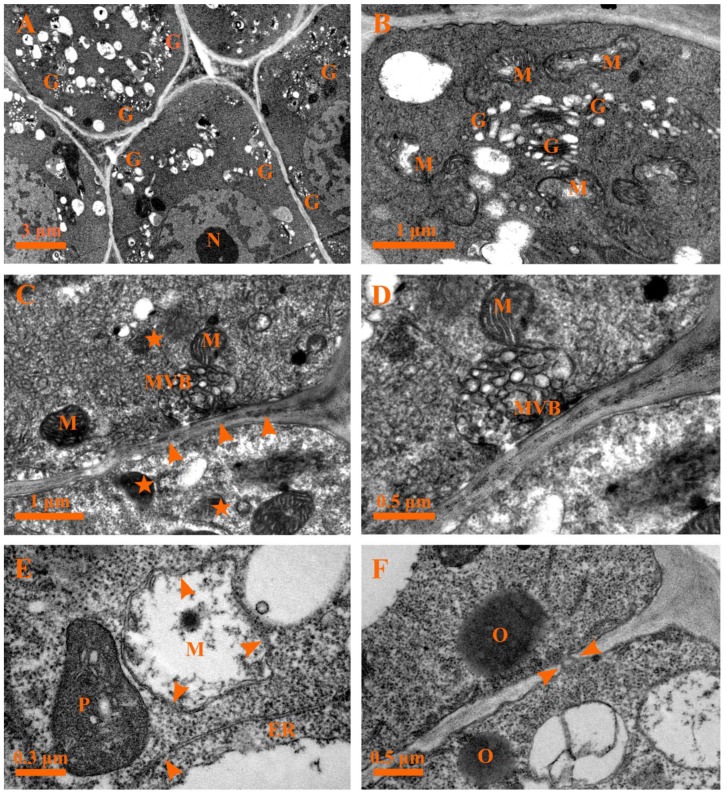
Ultrastructure of the capitate glandular hair in *D. dasycarpus*. (**A**) Late stage 3, cytoplasm condensation and shrinkage of the secretory cells and plenty of Golgi bodies. (**B**) Late stage 3, detailed Golgi bodies and deformed mitochondria. (**C**) Late stage 3, numerous small vesicles and myelin-like structures (asterisks) in the cytoplasm, plenty of dark osmiophilic substances in the intercellular space (arrowheads), and the multivesicular body fusing with plasma membranes and releasing the vesicles into the apoplast. (**D**) Late stage 3, detail of the multivesicular body fusing with plasma membranes. (**E**) Late stage 3, condensed plastid with fuzzed membrane, degraded mitochondrion and endoplasmic reticulum with a diffused end (arrowhead). (**F**) Late stage 3, showing oil droplets in the cytoplasm and plasmodesma (arrowheads). ER, endoplasmic reticulum; G, Golgi body; M, mitochondria; MVB, multivesicular body; N, nucleus; P, plastid; V, vacuole.

During the following developmental process, the cytoplasms of the secretory cells showed more significant condensed electron density and shrinkage (Figure 8A). The most striking ultrastructural features of late stage 3 were an evident increase in the number of Golgi bodies and the release of several vesicles from the Golgi bodies observed in the cytoplasm (Figure 8A,B). In addition, the multivesicular body (MVB) fused with the plasma membrane and released the vesicles into the cell wall (Figure 8C,D); numerous small vesicles and myelin-like structures coupled with various degenerated organelles were detected in the cytoplasm (Figure 8C), and the intercellular space contained plenty of oil droplets or dark osmiophilic substances during this time (Figure 8C). Meanwhile, degraded mitochondria (Figure 8B–E), end-diffused endoplasmic reticula, and electron-dense plastids with disorganized membrane systems (Figure 8E) were apparent at this stage. In addition, plasmodesma and the round-like oil droplets or electron-dense osmiophilic substances were observed (Figure 8F).

#### 2.5.4. Stage 4: Late Degeneration Stage

**Figure 9:**
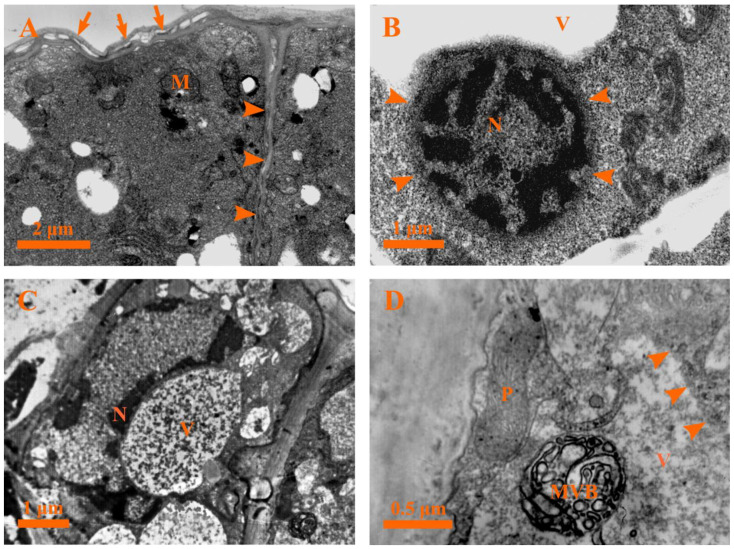
Ultrastructure of the capitate glandular hair in *D. dasycarpus*. (**A**) Stage 4, loosened cuticle layer (arrows), swollen and twisted cell walls (arrowheads), and dark osmiophilic substances in the head of glandular hairs. (**B**) Stage 4, degraded nucleus with condensed chromatin and diffused membrane (arrowheads) in the head cell. (**C**) Stage 4, misshapen nucleus with heterochromatin attached to the nuclear envelope in the stalk cell. (**D**) Stage 4, MVB in a degenerated vacuole of the stalk cell, showing a degraded vacuole membrane (arrowheads). M, mitochondria; MVB, multivesicular body; N, nucleus; P, plastid; V, vacuole.

**Figure 10:**
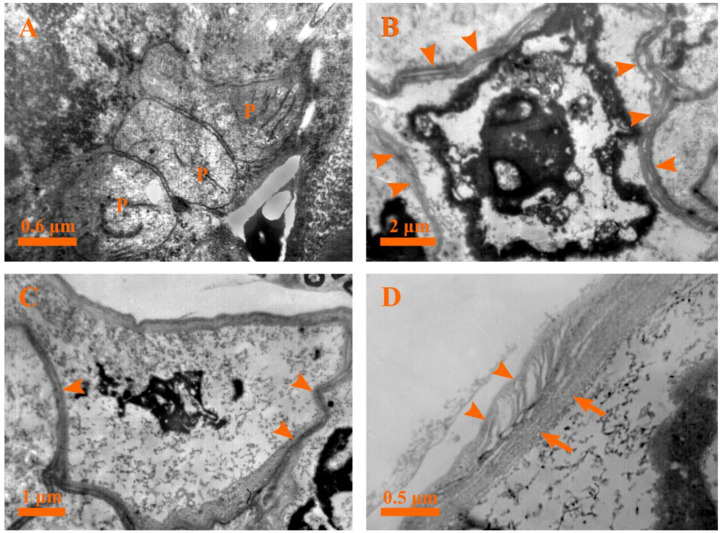
Ultrastructure of the capitate glandular hair in *D. dasycarpus*. (**A**) Stage 4, degraded plastids in the disorganized cytoplasm of the stalk cell. (**B**) Late stage 4, secretory cells with condensed and disorganized cytoplasms and loose walls (arrowheads). (**C**) Late stage 4, degraded cell wall (arrowheads) and minute dark residue in degraded secretory cells. (**D**) Late stage 4, degenerated cell wall (arrows) and cuticle (arrowheads). P, plastid.

With further development, the cuticle layer in the head of the glandular hair was loosely structured, the cell wall was swollen and twisted, and the membrane system of various organelles buckled or degraded and could not be effectively distinguished in the condensed cytoplasm (Figure 9A). The nucleus was identified by condensed chromatin and a diffused membrane (Figure 9B). Meanwhile, the stalk cell was characterized by a misshapen nucleus with visible heterochromatin tightly attached to the nuclear envelope (Figure 9C). Subsequently, the MVB and degraded plastids with degraded membranes were detected in the degraded vacuoles and disorganized cytoplasms, respectively (Figure 9D and Figure 10A).

Ultimately, the cytoplasms of the head cells appeared to be further disorganized, the plasma membrane of the head cells disintegrated (Figure 10B), and masses of condensed cytoplasm with round-like oil droplets or electron-dense osmiophilic substances and loose walls could be observed (Figure 10B). Ultimately, the protoplasts of the secretory cells degraded extensively, leaving minute irregular dark residues (Figure 10C), degenerated cell walls (Figure 10C), and degraded cuticles (Figure 10D).

## 3. Materials and Methods

### 3.1. Plant Materials

*Dictamnus dasycarpus* Turcz. is a perennial medicinal plant in the genus of *Dictamnus* L. in the Rutaceae family. For this study, *D. dasycarpus* materials were collected from the Qinling Mountains in Shaanxi, China (33°59′31″ N, 108°58′13.6″ E), and very young leaves and inflorescences were collected from the vegetative to reproductive stages (May to July). Our field studies were conducted in accordance with the local legislation, and appropriate permissions were obtained.

### 3.2. Light Microscopy

*D. dasycarpus* leaves, ovaries, floral axes, and filaments were cut into 1–2 mm^3^ pieces and fixed in 2.5% glutaraldehyde in 0.1 M phosphate buffer (pH 7.0) at 4 °C for 4 h. After three 30-min rinses in 0.1 M phosphate buffer, pH 7.0, the samples were post-fixed overnight at 4 °C in 1% osmium tetroxide. Then, after three 30-min rinses in 0.1 M phosphate buffer, pH 7.0, they were rinsed three times with redistilled water. The samples were dehydrated in a gradient ethanol series (30%, 50%, 70%, 85%, and 90% once each, then twice in 100%), and then embedded in Epon 812. Successive semi-thin sections (1–2 μm) were cut using a Reichert-Jung ultramicrotome and stained with toluidine blue O [15]. The sections were examined and digitally recorded using a Leica microscope (DMLB) equipped with a video camera (DFC 7000T; Wetzlar, Germany). Furthermore, a Leica EZ 40 stereo light microscope was also used in the examination.

### 3.3. Scanning Electron Microscopy

Samples from the *D. dasycarpus* inflorescences were fixed in 2.5% glutaraldehyde for 4 h, sequentially washed in phosphate buffer, and post-fixed in 1% osmium tetroide. After three rinses with phosphate buffer, pH 7.0, the samples were dehydrated using a graded ethanol series and dried using an Emitech K850 critical point dryer (Quorum Emitech, East Grinstead, West Sussex, UK). The samples were then coated with gold on a Hitachi E-1010 Ion sputter-coater (Hitachi High-Technologies Corporation, Tokyo, Japan) and examined using a Hitachi S570 scanning electron microscope [20].

### 3.4. DAPI and TUNEL Assays

DNA fragmentation during the PCD process can be specifically detected using TUNEL, a terminal deoxynucleotidyl transferase-mediated dUTP nick-end labeling reaction [15]. To assay DNA degeneration, the samples were fixed, dehydrated, embedded in wax, and sliced into 8 μm sections using a Leica RM 2135 rotary microtome, dewaxed twice in xylene for 20 min each, and then rehydrated through a graded ethanol series (in 100% twice, and then once each in 90%, 85%, 70%, and 50%). After rinsing three times with 0.1 M phosphate buffer, pH 7.4, the sections were soaked in Proteinase K (10 μL 10× Proteinase K in 90 μL of 0.1 M phosphate buffer, pH 7.4) in a humid chamber for 30 min at 37 °C, followed by three rinses with phosphate-buffered saline. The in situ nick-end labeling of nuclear DNA fragmentation was performed for 60 min at 37 °C using a TUNEL apoptosis detection kit (KeyGen Biotech, Nanjing, China), according to the manufacturer’s instructions. For each experiment, one positive control was treated with DNase I (60 μL of 3000 U Dnase I + 40 μL of DNase I Buffer) for 30 min at 37 °C before labeling. One negative control was included without the terminal deoxynucleotidyl transferase (TdT) enzyme.

The TUNEL-labeled sections were washed three times in 0.1 M phosphate buffer before being stained in the dark for 30 min at 37 °C with 2 mg L^−1^ DAPI in 10 mL of dilution buffer (Bioworld Technology, Nanjing, China). Finally, all of the sections were washed with phosphate-buffered saline. The nuclei were observed after excitation at 450–500 nm and 340–380 nm using a Leica DMLB epifluorescence microscope, and then photographed using a Leica microscope (DMLB) equipped with a video camera (DFC 7000T; Wetzlar, Germany) for TUNEL detection and DAPI, respectively.

### 3.5. Viability Staining—Evans Blue

Samples from the *D. dasycarpus* leaves and inflorescences were stained in 0.1% Evans blue dissolved in dH_2_O for 30 min [21]. The stained samples were then washed in distilled water and photographed using a Leica EZ 40 stereo light microscope and a Leica microscope (DMLB) equipped with a video camera (Leica DFC 7000T).

### 3.6. Transmission Electron Microscopy

The samples were treated the same as for light microscopy. After embedding in Epon 812, the ultra-thin sections obtained using a Leica EM UC 6 ultramicrotome were stained with uranyl acetate [22] and lead citrate [23] and observed using an H-600 TEM (Hitachi, Japan) at 75 kV.

## 4. Discussion

### 4.1. Origin and Differentiation

**Figure 11:**
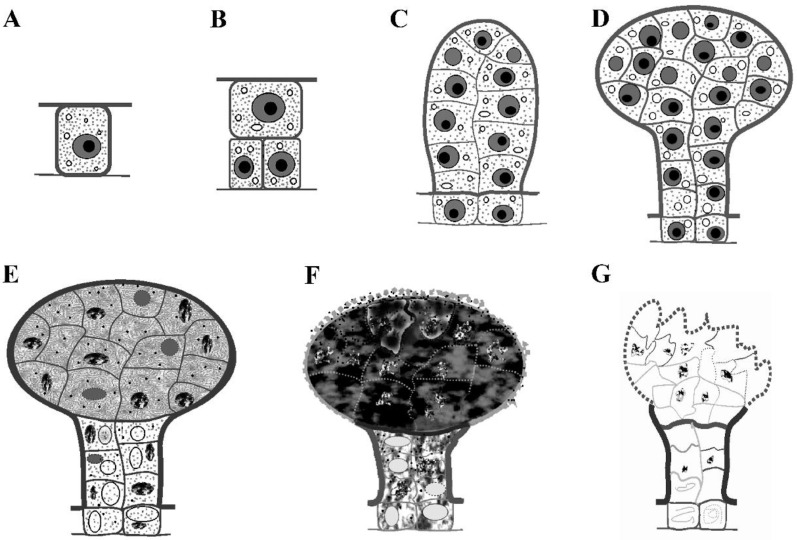
Semi-schematic drawings for development of the capitate glandular hairs. (**A**) Stage 1, one initial cell. (**B**) Stage 1, anticlinal division of the lower cell. (**C**) Stage 1, a cylindrical shape with several cells formed. (**D**) Stage 2, head and stalk of the capitate glandular hair. (**E**) Early stage 3, condensed cytoplasm occurred in the multicellular head. (**F**) Late stage 3, condensed cytoplasm occurred in the multicellular head and stalk cells with disorganized cytoplasms. (**G**) Late stage 4, degenerated head cells with little residues and evident cutinization of the stalk cells.

Rutaceae is characterized by secretory cavities, which have received great interest for their essential oils and formation mode [3,6,13,15]); however, the capitate trichomes in this family have rarely been involved. *D. dasycarpus* is characterized by two types of glandular hairs: trichome-like cavity and capitate glandular hair. This study demonstrates the capitate glandular hair origin by one single epidermal cell that differentiated into a typical capitate glandular hair with two basal epidermal cells, two cell-lined stalks, and a multicellular head (Figure 11). Furthermore, the capitate glandular hair origin is homologous with the trichome-like cavity, which is characterized by the combined characteristics of non-glandular hair, capitate glandular hair, and a secretory cavity in *D. dasycarpus* [20], indicating a potential evolutionary relationship between these glands and non-glandular hairs. However, detailed work is still required in further studies.

### 4.2. Development Process of PCD

Recently, several cases of PCD have been conducted in plant secretory structures, and the degeneration characteristics conducted in the secretory cells have shown different patterns in different types of glands [11,12,14,15,17,18,19,20]. The present study describes the morphology, cellular ultrastructure, and biochemical aspects of the glandular hairs in *D. dasycarpus* from a developmental perspective. During this compelling and systematic process, the nucleus exhibits DNA fragmentation, chromatin condensation, and nuclear membrane disruption. In addition, the protoplast undergoes cytoplasmic fragmentation, degradation of the plastid and mitochondrion, diffusion of the ends of the endoplasmic reticulum, plasma membrane rupture, cell wall degradation, and cuticle dilation, demonstrating a typical developmentally regulated PCD involved in the formation of the glandular hairs in *D. dasycarpus*.

#### 4.2.1. Loss of Cell Viability

The plasma membrane often degrades during plant PCD, losing integrity and, consequently, viability [21,24]). Evans blue stains only those cells with degraded plasma membranes, indicating the loss of membrane integrity and viability [21]. In this study, Evans blue staining was applied to better characterize the viability of the secretory cells in the capitate glandular hairs of *D. dasycarpus.* The results showed that the head and stalk cells of the capitate glandular hairs stained with Evans blue were identified by a positive reaction, and this was ultrastructurally consistent with the degraded plasma membrane at the late stage. Plasma membrane degradation is always observed in the PCD of trichome-like cavities in *D. dasycarpus* [20], wheat endosperm [21], and lace plant leaf formation [24]. Moreover, the loss of cell viability in this gland seems orderly, as it is observed first in the head and then in the stalk cells, which is consistent with the results from the light microscope.

#### 4.2.2. Cytoplasm Condensation

In *D. dasycarpus*, the most significant events in the capitate glandular hair are cytoplasm condensation and shrinkage in stage 3, which are considered to be typical early symptoms of PCD in plant glands [2,11,13,17,25] and other plant tissues [26,27,28,29]. At a more advanced differentiation stage, the cytoplasm and membrane systems of most organelles in the capitate glandular hair of *D. dasycarpus* showed evidently disorganized characteristics and, subsequently, complete degradation.

#### 4.2.3. Differentiation of the Cell Walls

Previous studies on the glandular trichomes of *Prostanthera ovalifolia* demonstrated that the secretory cells lyse in fully mature glands [30]. In the present study, cell wall buckling with a wave-like shape was identified in the head cells of the capitate glandular hair of *D. dasycarpus* during stage 3, coupled with further cell wall collapse, which was observed in the PCD process of the pigment gland in *Gossypium hirsutum* [12], floral nectary in *D. purpurea* [11], trichome-like cavities in *D. dasycarpus* [20], cavities in *D. dasycarpus* [15] and *C. sinensis* [13,31], and floral nectary in *I. purpurea* [16]. At the late degeneration stage, intercellular spaces enlarged considerably, and the walls of the head cells became compressed and collapsed, coupled with cuticle disruption in the capitate glandular hairs of *D. dasycarpu*s, exhibiting typical features consistent with PCD. However, in plant PCD, the cell walls are always modified to a thicker shape in the sheath cells of the trichome-like cavities in *D. dasycarpus* [20], the mucilage cells of *A. angustifolia* [18], and tracheal elements [32]. In this study, cell wall thickening in the stalk was identified in the degenerated glandular hairs of *D. dasycarpu*s, as they might play a role as a mechanical defense to inhibit the invasion of pathogens or insect invasions [11].

#### 4.2.4. Degeneration of Organelles

Previous research has shown that ontogenesis in various types of plant glands is a PCD phenomenon in which different kinds of organelles degrade [13,15,18,20]. In the present study, the plastids and the mitochondria showed a condensed, degenerated thylakoidal system and a misshapen shape, respectively, associated with the ultimate disorder and degeneration of the membrane system. Furthermore, according to the DAPI and TUNEL assays, the nuclei of the glandular hair secretory cells were identified by their misshapen shapes and TUNEL-positive reactions during stage 3 and early stage 4. In addition, they were ultrastructurally identified by their deformed shapes with condensed chromatin and diffused membranes, which are considered to be typical features of PCD in plants [33]. This was also observed in the PCD process of *D. purpurea* floral nectary [11], *G. hirsutum* pigment glands [12], *D. dasycarpus* trichome cavities [20], and secretory cavities in *C. sinensis* [13,14,34] and *D. dasycarpus* [15].

#### 4.2.5. Autolysis and Autophagy

**Figure 12:**
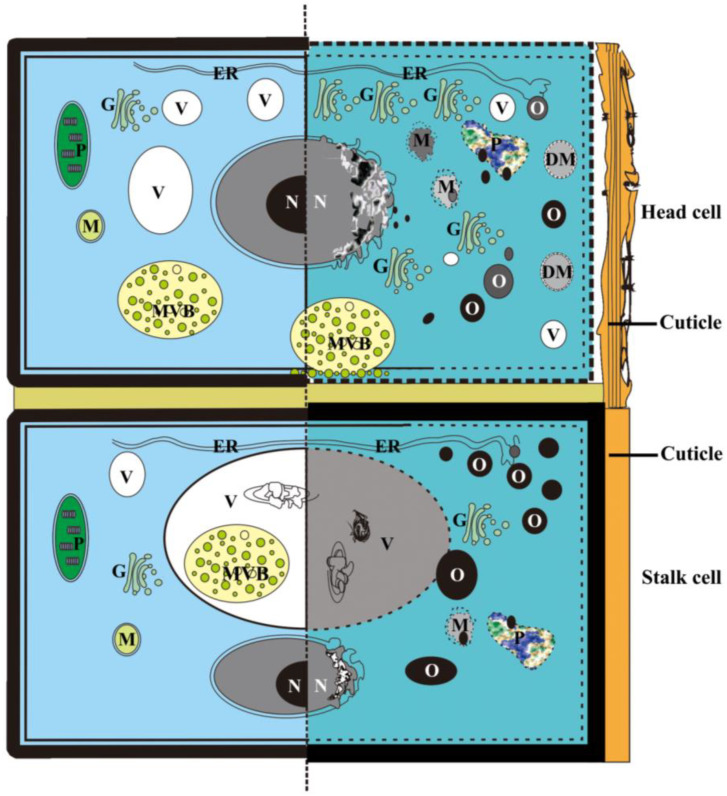
Schema for programmed cell death and secretion mechanism of the capitate glandular hair in *D. dasycarpus.* The head cell with the degraded cell walls and no evident vacuolization of the cytoplasm, as well as the stalk cell with thickening of the walls and evident vacuolization of the cytoplasm during the PCD process at the late stage, during which the oil droplets synthesized. DM, double membrane-bounded structure; ER, endoplasmic reticulum; G, Golgi body; M, mitochondria; MVB, multivesicular body; N, nucleus; O, oil droplet; P, plastid; V, vacuole.

Multilamellar bodies and double membrane-bound autophagosomes have been associated with PCD processes in several species [12,13,15,20,35,36]. In the present study, the multivesicular body in the cytoplasm seemed to fuse with the plasma membrane, releasing vesicles into the buckling cell wall, which was also observed in PCD during floral nectary senescence in *I. purpurea* [16], aerenchyma formation in *Typha angustifolia* leaves [37], and rhytidome and interxylary cork formation of *Astragalus membranaceus* [38] and other plant systems [39]. Interestingly, the fully developed head cells in the PCD process of *D. dasycarpu*s were not strongly vacuolated, and various organelles gradually degenerated, mainly in the cytoplasm, indicating non-autolytic PCD [33]. Furthermore, double membrane-bound structures were also detected in the disorganized cytoplasms, indicating an autophagic PCD [33]. Meanwhile, in the stalk cells, the MVB was detected in the degraded vacuoles. A degraded nucleus with condensed chromatin and diffused membrane, vacuolization, and eventual loss of the vacuole membrane integrity (Figure 12) demonstrated the vacuole-mediated autolytic PCD mechanism [33].

### 4.3. Hypothesis of the Essential Oil Synthesis

Most types of secretory structures, such as secretory cavities in Rutaceae and glandular hairs in Labiatae, are important sites for essential oil accumulation. The synthesis and accumulation of the essential oils have been widely conducted, and the cell matrix, plastids, mitochondria, endoplasmic reticula, and Golgi bodies are reportedly involved in oil synthesis in different species [6,40,41,42,43]. In *D. dasycarpus*, senescence of the capitate glandular hairs demonstrated a typical developmentally regulated PCD process, during which the essential oils or numerous micro-dark osmiophilic substances were observed in the disorganized cytoplasm, coupled with the autolysis of various organelles or protoplast degradation (Figure 12). Meanwhile, several cases of the cellular constituent degeneration—including electron-dense plastids, degraded mitochondria, end-expanded endoplasmic reticulum, and buckling cell walls—were reported in this study before evident secreting activities, which were observed in the senescent trichomes of *Achillea millefolium* [44], the floral nectary of *Arabidopsis thaliana* [4], the PCD of laticiferous canals in fruits of *D. fargesii* [17], and secretory cavities in *D. dasycarpus* [15]. In addition, many Golgi bodies with numerous vesicles were found in the disorganized cytoplasm during stage 3, indicating a high level of secretion activity [45]. These were also observed during the PCD process of mucilage cells in *A. angustifolia* [18]. Furthermore, studies have confirmed that plant cells always remain viable during the majority of the developmental program leading to cell death [46]. Dark oil droplets and numerous micro-dark osmiophilic substances were identified in the degraded cytoplasm and intercellular space, while no organelles could be observed at the late stages. In a previous study on the secretory cavities of *D. dasycarpus*, we discovered that after the degeneration of the center secretory cells, an abundance of oil droplets accumulated in the outer secretory cells [15]. Furthermore, degradation products were proven to construct secondary cell walls during tracheary element differentiation [45]. Both of these phenomena were confirmed as PCD processes [15,47].

## 5. Conclusions

In this study, we speculated that senescence of the capitate glandular hairs in *D. dasycarpus* could be a typical developmental PCD in plants, during which essential oils or numerous micro-dark osmiophilic substances are observed in the disorganized cytoplasm, coupled with the autolysis of various organelles or protoplast degradation. Furthermore, a close relationship between secretory cell degeneration and the synthesis of essential oils may exist in the glandular trichome of *D. dasycarpus*.

### Future Directions

In plant glands, significant progress has been made in identifying the distinct mechanisms of PCD. Due to their differing biological functions and secretions, different types of secretory structures have shown distinct characteristics during the development and senescence processes, and it is intriguing that different modes of cell death always co-exist in one type of plant gland. This observation could lead to a better understanding of plant crosstalk. Further studies on the development and secretion process of different plant glands to further explore the biosynthesis pathways of plant secondary metabolites will be of great biological significance.

## Data Availability

Not applicable.
